# Water can trigger nuclear reaction to produce energy and isotope gases

**DOI:** 10.1038/s41598-023-50824-8

**Published:** 2024-01-02

**Authors:** Bin-Juine Huang, Yu-Hsiang Pan, Po-Hsien Wu, Jong-Fu Yeh, Ming-Li Tso, Ying-Hung Liu, Litu Wu, Ching-Kang Huang, I-Fee Chen, Che-Hao Lin, T. R. Tseng, Fang-Wei Kang, Tan-Feng Tsai, Kuan-Che Lan, Yi-Tung Chen, Mou-Yung Liao, Li Xu, Sih-Li Chen, Robert William Greenyer

**Affiliations:** 1 Chair Professor, Vehicle and Energy Engineering Program, National Taiwan Normal University, Taipei, Taiwan; 2 Advanced Thermal Devices (ATD), Inc., Konglin Group, New Taipei City, Taiwan; 3 Mastek Technologies, Inc., New Taipei City, Taiwan; 4 Institute of Nuclear Engineering and Science, National Tsing Hua University, Hsinchu, Taiwan; 5 Department of Mechanical Engineering, University of Nevada, Las Vegas, NV USA; 6 Department of Mechanical Engineering, National Taiwan University, Taipei, Taiwan; 7 Martin Fleischmann Memorial Project, Worthing, UK

**Keywords:** Energy science and technology, Engineering

## Abstract

This paper reports the discovery that water can trigger a peculiar nuclear reaction and produce energy. Cavitation may induce unusual reactions through implosion of water vapor bubbles. Many of this research has been published formally or informally. We have conducted experiments using two reactor types made from multiple-pipe heat exchanger and found that the heat exchange process of water produces peculiar excess heat and abnormally high pressure leading to rupture of the reactor. Recently, we have tested another eight reactors. Interestingly, these reactors produce non-condensable gas. We suspected that they include ^22^Ne and CO_2_. We used a mass spectrometer (MS) to analyze 14 gas samples collected from 8 reactors, including ten samples showing a coefficient of performance COP_x_ > 1.05 (with excess heat) and four having COP_x_ < 1.05 (without excess heat). Several methods were adopted to identify the gas content. For CO_2_ identification, two methods are employed. For ^22^Ne identification, three methods are employed. All the results confirm that isotope ^22^Ne and regular CO_2_ really exist in the output gas from reactors determined to have excess heat. We conjecture a possible mechanism to produce ^22^Ne and CO_2_ and find out that ^12^C and isotope ^17^O are the intermediate. They finally form isotope gases containing ^17^O, including H_2_O-17 (heavy-oxygen water), isotope O_2_ (^16^O–^17^O), and isotope CO_2_ (^12^C–^16^O–^17^O). In the excess heat producing reactors, all these gasses were detected by MS in the absence of ^20^Ne and ^21^Ne. The observed isotope gases produced from reactors having excess heat verifies that water can trigger a peculiar nuclear reaction and produce energy.

## Review of peculiar phenomena observed in heat exchange process of water

Possible energy production via water cavitation has been noted for a long time. It was occasionally reported formally or informally that cavitation of water may induce some form of reaction by way of implosion of water vapor bubbles which produces excess energy^1–10^.

We have conducted experiments using two reactors made from concentric multiple-pipe heat exchanger and found that, when water is flowing through a tiny space and heated, it produces peculiar excess heat probably by cavitation and dynamic implosion of nanobubbles^11^. Water used in the experiments is the city water filtered by reverse osmosis (RO) filter.

The first reactor (VCS)^11^ is a triple-pipe heat exchanger (THX) (about 30 meter long) using R22 vapor from a freon compressor (3 kW input) as the heat source to heat the pressurized water (about 21 bar) flowing through a tiny channel of THX, about 2 mm gap. The water flow can be controlled as a pulse flow, about 2 to 10 cycles per minute, through a control valve. VCS was developed for 2 years with several modifications^11^. The inlet water temperature varied between 10 and 55 °C at average flowrate around 1.2 liter/min. The compressor outlet temperature varied around 150–160 °C. Modification of VCS-1, VCS-2a, VCS-2b, VCS-2c, VCS-3 includes the change of pulse cycle period of water flow, the optimization of piping resistance in THX, and the change of lubrication oil of compressor which will alter the heating rate of water inside THX.

**Figure 1:**
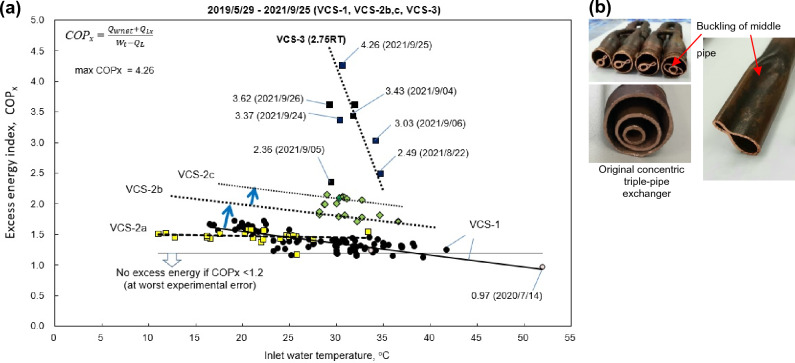
Test results of Reactor 1 (VCS). (**a**) Variation of reactor’s COP_x_ during modifications [adding extended data of Ref.^11^]. (**b**) Buckling and deformation of pipes in VCS-1 when COP_x_ > 2.0. [from Ref.^11^].

The coefficient of performance COP_x_ is defined as the ratio of heat output to heat input across the reactor at steady state, (Q_wnet_ + Q_Lx_)/(W_t_ − Q_L_). The maximum COP_x_ obtained in VCS was 4.26, Fig. 1a. Some peculiar phenomena were observed in VCS during the tests. Abnormally high pressure (greater than 720 bar) was observed which ruptured the pressure gauges and copper pipes, Fig. 1b. Possible nuclear transmutation was found by SEM/EDX inspection of ruptured copper pipe samples (C increases 200–500%, O 300–600%, Fe 400%, and new elements P, S, Ca appears).

**Figure 2:**
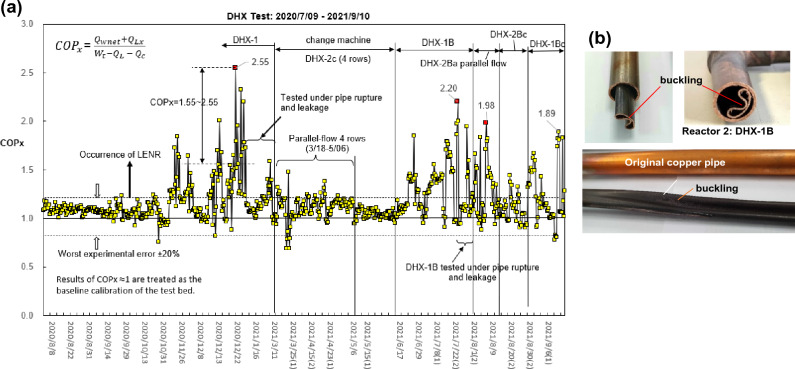
Test results of Reactor 2 (DHX). (**a**) Variation of reactor’s COP_x_ during modifications [adding extended data of Ref.^11^]. (**b**) Pipe rupture in DHX-1B when COP_x_ > 2.0 [from Ref.^11^].

The second reactor (Reactor 2) is a double-pipe heat exchanger (DHX)^11^. The pulsed water flow is heated inside the DHX by hot steam from a boiler. Shown in Fig. 2a is the performance variation during the development^11^. The maximum COP_x_ obtained was 2.55. Similar pipe rupture due to extreme high pressure (greater than 240 bar) takes place when COP_x_ > 2.0. Possible nuclear transmutation in ruptured copper pipe was also observed, Fig. 2b. It was found that C increases 300%, O increases 700–800%, and Cl increases 63%.

## Non-condensable gases found in new reactors and analyzed

**Figure 3:**
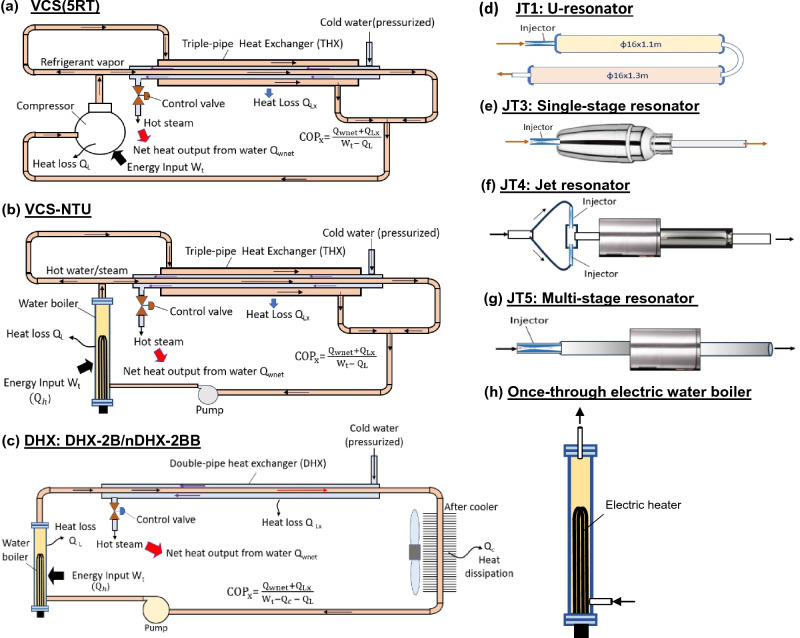
Reactors tested in the present study. (**a**) VCS(5RT): THX heated by freon from compressor; (**b**) VCS-NTU: THX heated by water boiler; (**c**) DHX: double-pipe heat exchanger heated by water boiler; (**d**) JT1: U-resonator; (**e**) JT3: single-stage resonator; (**f**) JT4: jet resonator; (**g**) JT5: multi-stage resonator; (**h**) once-through electric boiler to supply heat to reactors and act as a non-excess heat device. Reactors (d), (e), (f), (g) are connected to the water boiler (h) to run performance test.

Recently, we continued to develop new reactors using thicker material and simpler structure for preventing rupture and easy scale-up. Figure 3 shows the schematic diagram of eight different new reactor designs. The new reactors are all heated by a compact once-through electric water boiler, Fig. 3h, except VCS(5RT) in which THX is heated by a hot freon vapor from compressor. The resonator-type reactors have a simple structure for easy scale-up and may create a fluid resonance to enhance the cavitation effect. The heat exchange process between two streams inside the THX or DHX does not appear in the resonator-type reactors.

The design of new reactors is briefly described as follows:

- VCS(5RT): THX heated by 5 RT freon compressor and using stronger copper pipe.
- VCS-NTU: THX heated by once-through water boiler and using stronger copper pipe.
- DHX: same as Reactor 2 but having stronger copper pipe and with injector (nDHX).
- JT1 (U-resonator): U-shape pipes with injector.
- JT3 (Single-stage resonator): a single volume connected to injector.
- JT4 (Jet-resonator): jet impinging by two opposite injectors connected to a resonator.
- JT5 (Multi-stage resonator): multiple volumes connected in series.

**Figure 4:**
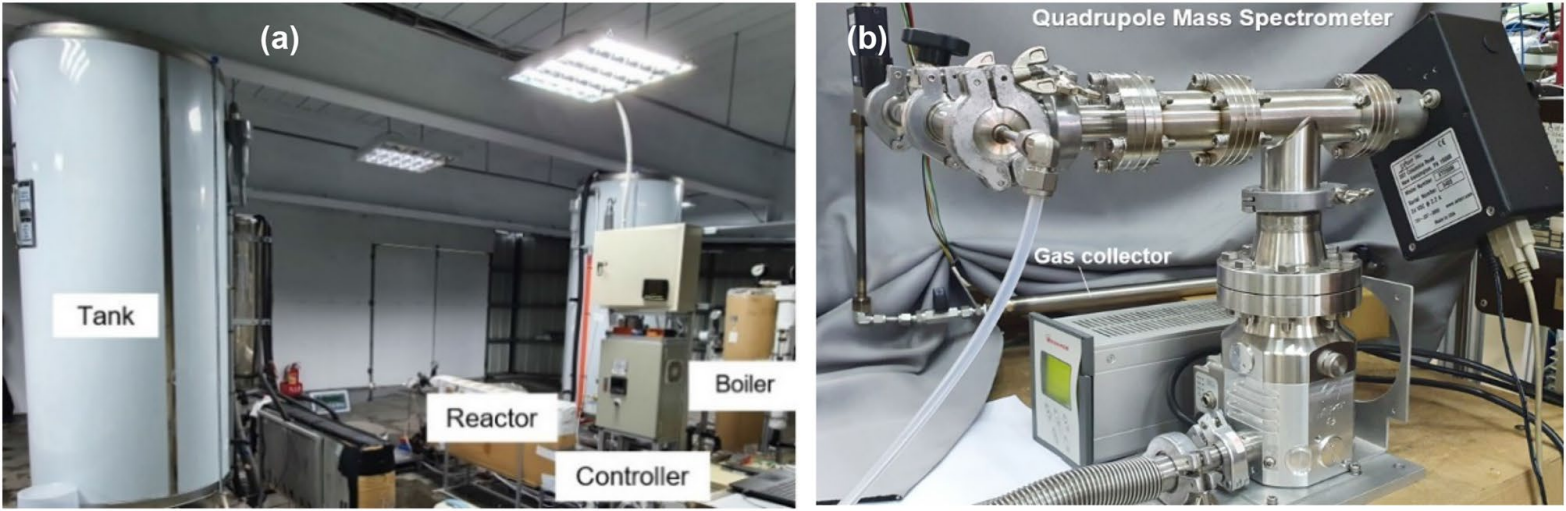
Test facility. (**a**) Performance test facility. (**b**) Gas collector and quadrupole mass spectrometer used.

We also developed a new test facility, Fig. 4a, which provides a maximum power input 10 kW to the boiler and supplies boiling water at maximum temperature 190 °C to the reactors. A RO equipment with 1000L storage tank is used to supply water.

Anomalous non-condensable gases were found during the test of the reactors. We designed a gas collecting unit to collect the output gas from the reactors for analysis. A mass spectrometer manufacturer (Mastek Co, Taiwan) and the experts team carried out the mass spectrometry. Two quadrupole mass spectrometers (model: Extorr XT200M and XT300M) were used interchangeably, as seen in Fig. 4b. The resolution of the mass spectrometer is better than 0.5 amu at 10% peak height and the minimum detectable partial pressure 5 × 10^−14^ Torr.

We collected gas samples from reactors running at steady state using the gas collector and then sent to manufacturer for spectrometry. The performance test was run at a steady state at least one hour to purge out the remaining impurities inside the reactor before collecting gas samples. The measuring of COP_x_ and thermal performance is the same as in Ref.^11^.

## Overall mass spectrum of sampled gases

**Figure 5:**
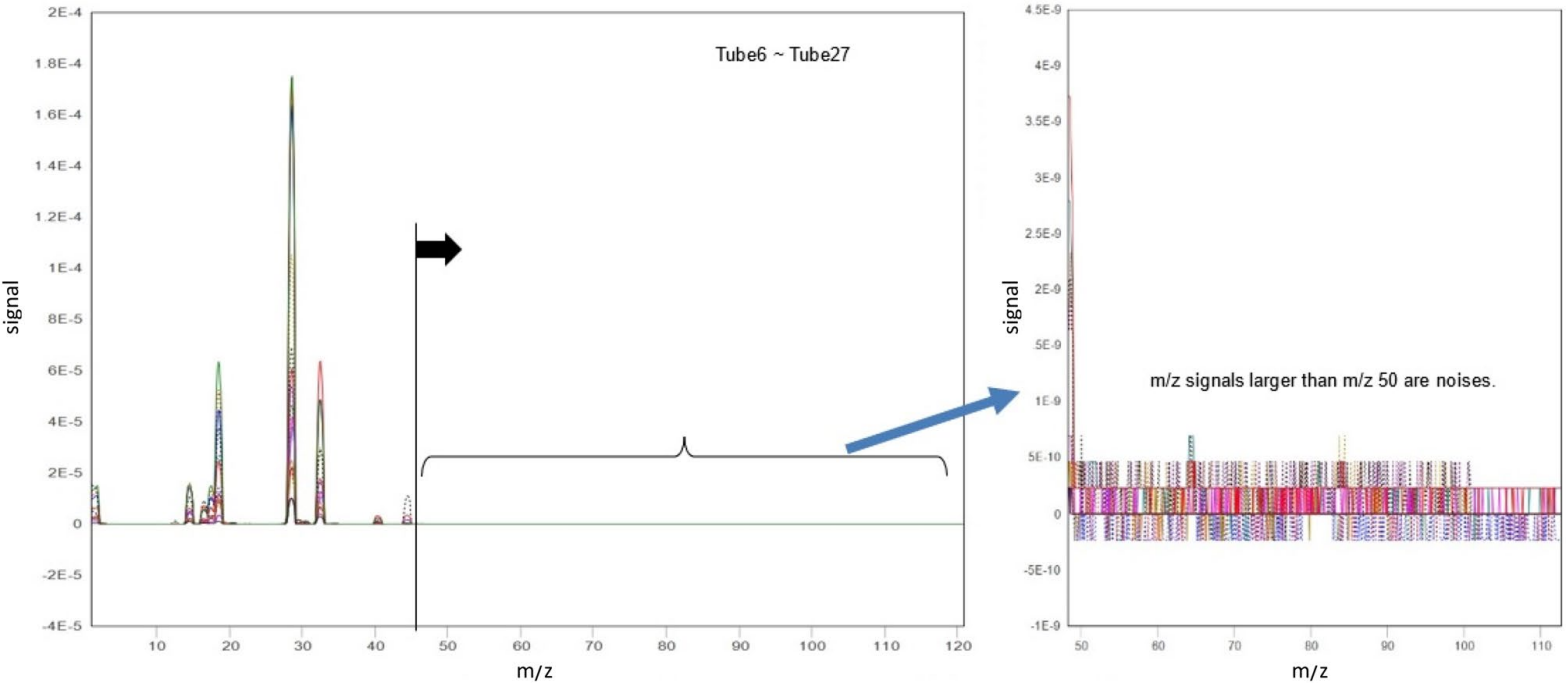
Overall mass spectrum of all the gas samples.

At the first batch, we collected 14 gas samples (named: Tube6-Tube27) from 8 reactors for mass spectrometry. It is very interesting that the overall mass spectrum of all the gas samples have no significant m/z signals at m/z higher than 50. This means that there are no high-mass compounds in the gas samples to produce interference on lower m/z signals. And all gas samples have similar mass spectrum except the signal intensity (Fig. 5). This makes the identification of gas content using mass spectrometry much easier.

## Identification of CO_2_ gas

Mass spectrometer (MS) was used to analyze 14 gas samples collected from 8 reactors, 10 samples having excess heat. Four gas samples (Tube9, 12, 17, 27) are from reactors without excess heat (COP_x_ < 1.05, considering the most-probable experimental error^11^), including Tube9 directly from the boiler (no reactor) as the reference. Tube12 and Tube27 are from failure reactor. Tube17 gas was collected during the test bed calibration using the reactor VCS-NTU under the condition of no excess heat. Table 1 lists the identification tag (ID) of gas samples and reactors of the first batch.

**Table 1:** Identification of CO_2_ presence using isotope ratio K44.

Reactor ID	Tube6	Tube7	Tube8	Tube9 (steam)	Tube10	Tube12 failure product	Tube13	Tube14	Tube 16	Tube17 baseline test	Tube 18	Tube 23	Tube 24	Tube27 failure product
Gas source (Reactor)	VCS (5RT)	VCS (5RT)	VCS (5RT)	only boiler	VCS (5RT)	JT1-n3S	DHX-2B	JT4-BV	DHX-2B	VCS-NTU(c)	JT3-CV	nDHX-2B	VCS-NTU	JT5-A5
m/z 44(gas) peak signal	4.46E−07	3.605E−06	1.89E−06	1.68E−08	7.50E−08	2.00E−08	1.50E−07	7.15E−07	1.97E−07	1.19E−07	1.90E−07	3.34E−07	1.09E−05	1.90E−07
m/z 44(air) peak signal	2.77E−07	2.857E−07	2.70E−07	1.79E−08	4.00E−08	5.10E−08	6.00E−08	5.50E−08	1.10E−07	8.01E−08	1.10E−07	1.39E−07	1.39E−07	1.39E−07
excess heat (COPx > 1.05)	Y	Y	Y	**n**	Y	**n**	Y	Y	Y	**n**	Y	Y	Y	**n**
Measured COPx	1.53	1.61	1.61	**1.0**	1.51	**1.02**	1.17	1.10	1.20	**1.02**	1.05	1.20	1.57	**1.03**
m/z 40(gas) peak signal	2.60E−06	3.30E−06	2.41E−06	1.24E−07	3.49E−07	1.76E−07	5.89E−07	4.96E−07	8.73E−07	6.08E−07	2.85E−07	1.34E−06	1.39E−06	1.29E−06
m/z 40(air) peak signal	2.64E−06	2.57E−06	2.57E−06	1.36E−07	4.41E−07	4.40E−07	5.21E−07	4.89E−07	9.54E−07	1.34E−07	1.37E−07	1.26E−06	1.26E−06	1.26E−06
I44(gas) = m/z 44(gas) ÷ m/z 40(gas)	0.17	1.09	0.78	0.14	0.22	0.11	0.25	1.44	0.23	0.19	0.67	0.25	7.85	0.15
I44(air) = m/z 44(air) ÷ m/z 40(air)	0.10	0.11	0.10	0.13	0.09	0.12	0.12	0.11	0.12	0.13	0.12	0.11	0.11	0.11
Internal standard ratio: K44 = I44(gas)/I44(air)	1.63	9.84	7.47	1.03	2.37	0.98	2.21	12.8	1.96	1.46	5.56	2.26	71.0	1.33
K44 > 1.5 (presence of CO_2_)	Y	Y	Y	**n**	Y	**n**	Y	Y	Y	**n**	Y	Y	Y	**n**

Significant values are in bold.

Two methods were employed to identify the presence of CO_2_ in gas samples. First, using the isotope ratio K44 defined with respect to the background air and internal standard based on m/z 40, we can identify the presence of CO_2_. The definition of K44 is K44 = I44(gas)/I44(air), where I44(gas) = m/z 44(gas) ÷ m/z 40(gas); and I44(air) = m/z 44(air) ÷ m/z 40(air).

It is very interesting to note from Table 1 that K44 are all lower than 1.50 for gas samples from reactors without excess heat (COP_x_ < 1.05). The high isotope ratio K44 (maximum 71.0 or > 5 mostly) in gases from reactors having excess heat strongly suggests the significant presence of CO_2_.

Another method to identify the CO_2_ existence in gas samples is to measure m/z 44 signal reduction of gas samples which has passed through a CO_2_ absorber Ca(OH)_2_ before entering the mass spectrometer. Pure CO_2_ gas, ambient air and MS blank were used as the reference. The rate of m/z 44 signal reduction, Ab44, for gas with (denoted as “Y”) and without (denoted as “n”) CO_2_ absorption is defined as: Ab44 = m/z 44(Y) ÷ m/z 44(n), and the reduction of CO_2_ (r_d_) is (1-Ab44)100%.

Each Ab44 is measured using the identical gas sample in a gas collector. It is seen from Table 2 that Ab44 of all the gases from reactors having excess heat is lower than 1.0 or m/z 44 signal reduction is between 36 and 80%. This is the direct proof of CO_2_ presence in gases from reactors having excess heat.

**Table 2:** Identification of CO_2_ presence from the reduction of m/z 44 signal caused by CO_2_ absorption.

Gas sample	Reactor	COPx	CO_2_ absorption by Ca(OH)_2_	m/z 44	Ab44 = m/z 44(Y) ÷ m/z 44(n)	Reduction ofm/z 44, r_d_ = (1 − Ab44)
Pure CO_2_	Reference gas	1.0 (No excess heat)	n	4.62E−04	0.019 (< 1)	92%
			Y	8.93E−06		
Atmosphere air	Reference gas	1.0 (No excess heat)	n	1.87E−06	1.14	n
			Y	2.14E−06		
MS blank	Reference gas	1.0 (No excess heat)	n	8.09E−06	0.98 (≈ 1.0)	n
			Y	7.96E−06		
Tube16	DHX-2B	1.20	n	1.99E−07	0.37 (< 1)	63%
			Y	7.47E−08		
Tube39	VCS-NTU	1.54	n	4.42E−06	0.74 (< 1)	36%
			Y	3.28E−06		
Tube40	VCS-NTU	1.21	n	6.70E−06	0.52 (< 1)	48%
			Y	3.51E−06		
Tube42	nDHX-2BB	1.22	n	1.68E−05	0.20 (< 1)	80%
			Y	3.40E−06		
Tube44	nDHX-2BB	1.16	n	9.98E−06	0.46 (< 1)	54%
			Y	4.62E−06		
Tube46	nDHX-2BB	1.15	n	5.52E−06	0.58 (< 1)	42%
			Y	3.20E−06		

## Identification of Neon gas ^21^Ne and ^20^Ne

**Figure 6:**
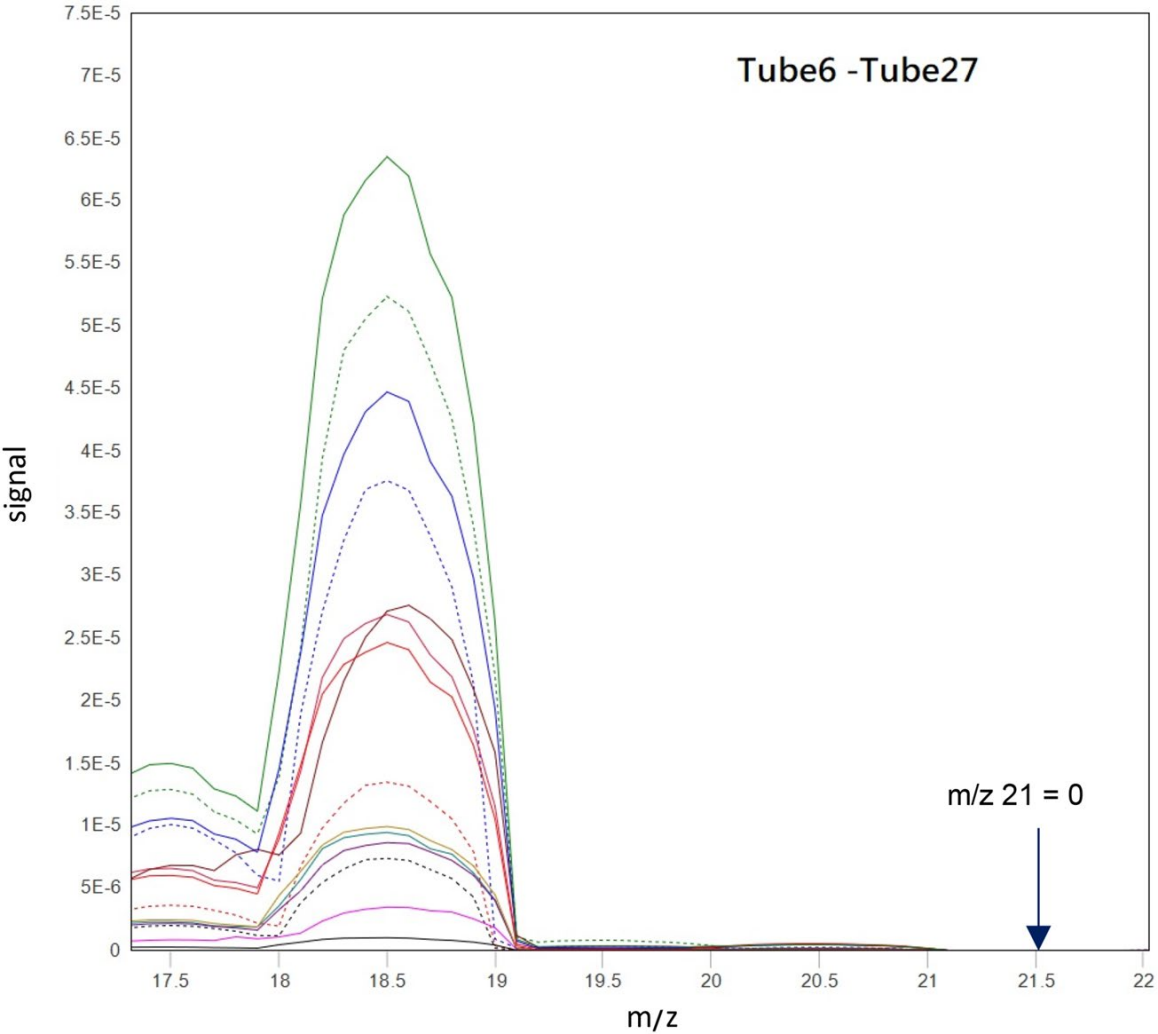
Mass spectrum of gas samples from m/z 17 to m/z 22 showing no m/z 21 signal at all.

Bob Greenyer suggested that Ne (^20^Ne, ^21^Ne, or ^22^Ne) may be produced if excess heat takes place^12^. First, we checked the possibility of ^21^Ne presence from the m/z 21 signal. It is seen from the mass spectrum of all gas samples shown in Fig. 6 that, no m/z 21 signal is present and hence ^21^Ne does not exist.

Since m/z 20 signal can be generated from natural abundance of Argon gas (m/z 40), the relative isotope ratio K20 (based on internal standard using m/z 40) is used to identify the presence of ^20^Ne by comparing with the background air. The definition of isotope ratio K20 is K20(gas) = I20(gas)/I20(Ar) where I20(gas) = m/z 20(gas) ÷ m/z 40(gas); I20(Ar) = m/z 20(Ar) ÷ m/z 40(Ar).

**Figure 7:**
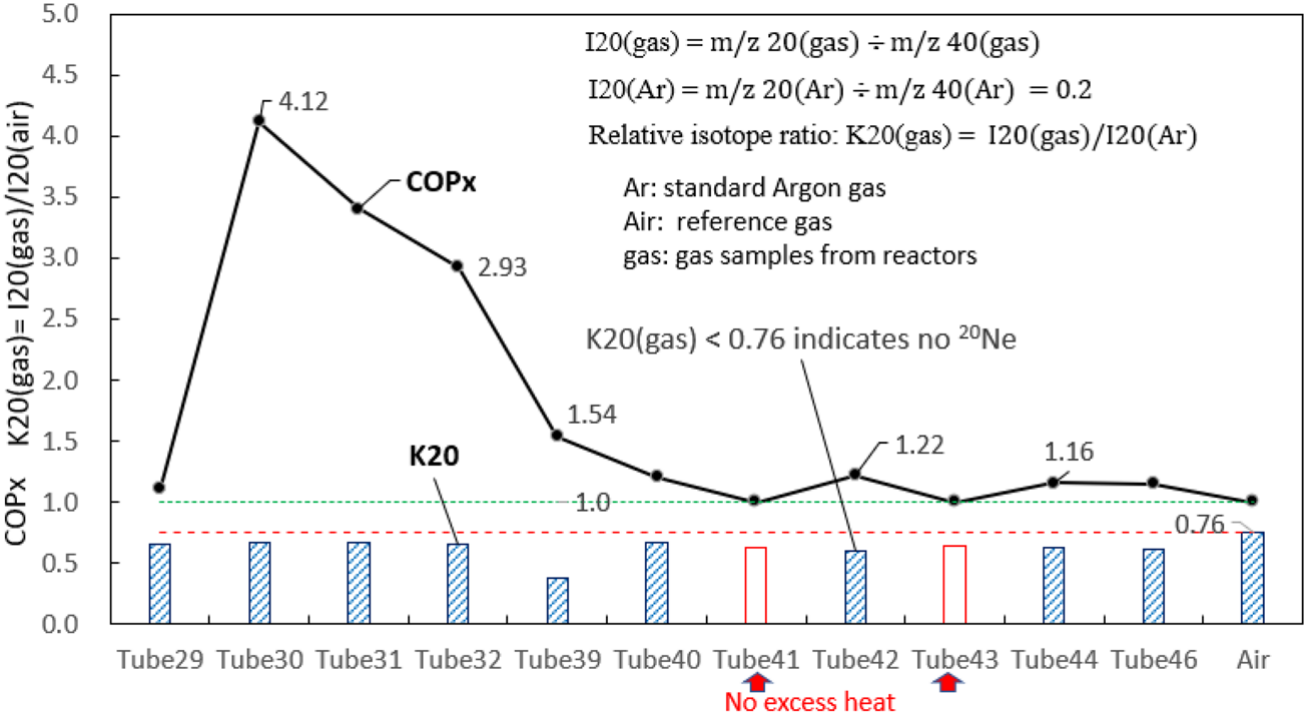
Variation of K20 for gas from different reactors. K20 < 0.76 indicates no ^20^Ne presence.

Pure Argon gas is used as the calibration gas whose K20(air) = 0.76. If K20(gas) < K20(air) = 0.76, it reveals that no ^20^Ne exists in gas sample. It is seen from Fig. 7 that K20 for all gas samples are smaller than 0.76. This confirms that no ^20^Ne is present in all gas samples, regardless of excess heat occurrence.

## Identification of Neon gas ^22^Ne

The presence of ^22^Ne in gases from reactors having excess heat were verified using three methods:

1. From isotope ratio K22 based on internal standard m/z 40.
2. From mathematics based on the observation of parameter G > 1 in gases from reactors having excess heat.
3. From isotope-ratio K24a and using CO_2_ absorber.

### Proof of ^22^Ne presence from isotope ratio K22 based on internal standard m/z 40

The m/z 22 signal is generated from ^22^Ne gas and CO_2_++ made by CO_2_ ionization in MS, while CO_2_ is the product of reactors having excess heat as described previously. The isotope ratio K22 is defined based on the internal standard m/z 40 as: K22 = I22(gas)/I22(air), where I22(gas) = m/z 22(gas) ÷ m/z 40(gas) and I22(air) = m/z 22(air) ÷ m/z 40(air).

**Figure 8:**
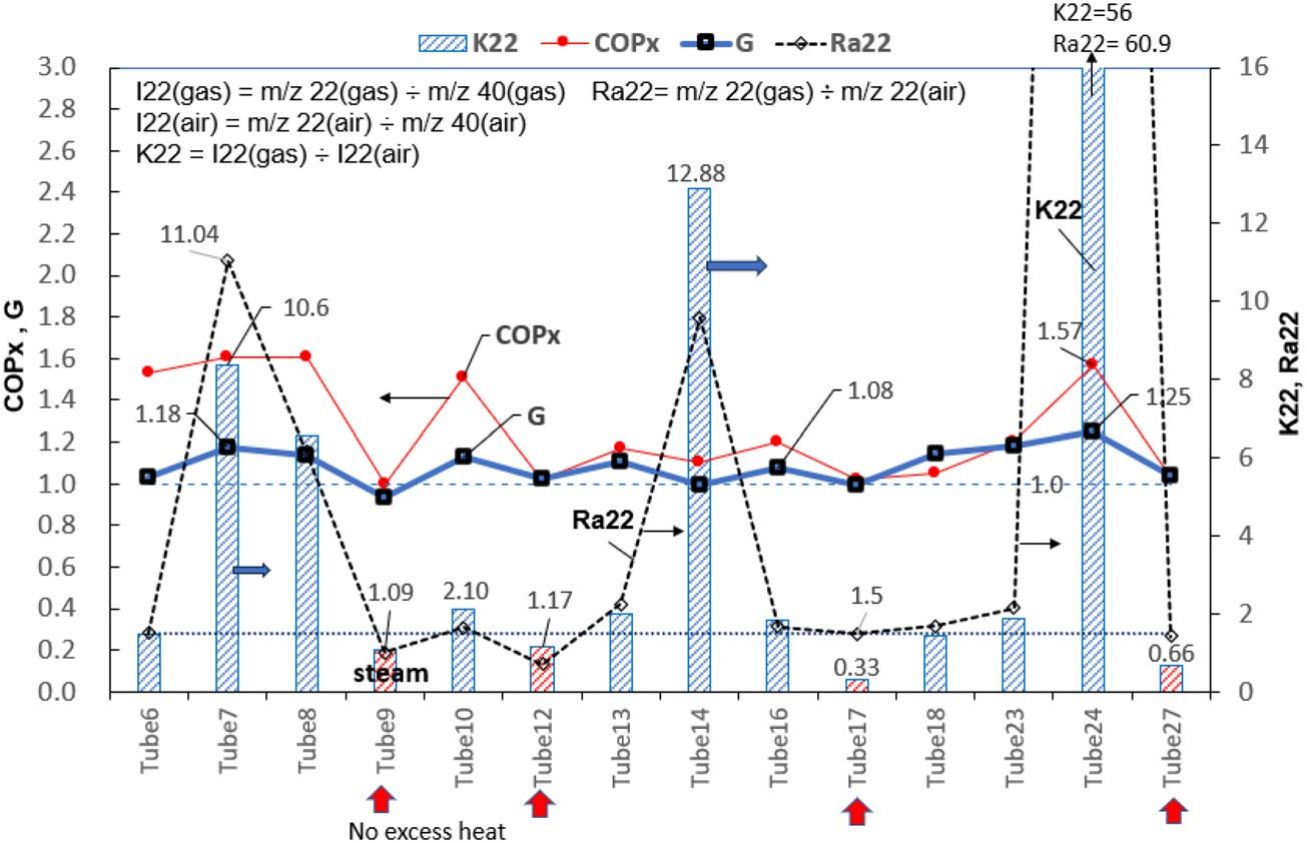
Variation of K22 for gas from different reactors. K22 > 1.5 indicates ^22^Ne presence.

Since the interference of CO_2_++ on m/z 22 signal is not very high, less than 2% of m/z 44 signal for pure CO_2_^13^, the isotope ratio K22 > 1.5 is beyond CO_2_++ interference in all gases from reactors having excess heat. The results shown in Fig. 8 suggests that m/z 22 signal contains those generated from ^22^Ne. Very high K22 (mostly higher than 2.0, highest 56.0) confirms that ^22^Ne is present in gases from reactors having excess heat.

### Proof of ^22^Ne presence from mathematics based on the observation of G > 1 in gases having excess heat

We found an interesting parameter G defined as G = R42(gas)/R42(air) where R42 = m/z 44 ÷ m/z 22, which is always greater than 1.0 in gases from reactors having excess heat (COP_x_ > 1.05). G can be determined from the measurement of m/z 22 and m/z 44 of gas and background air using the definition.

**Figure 9:**
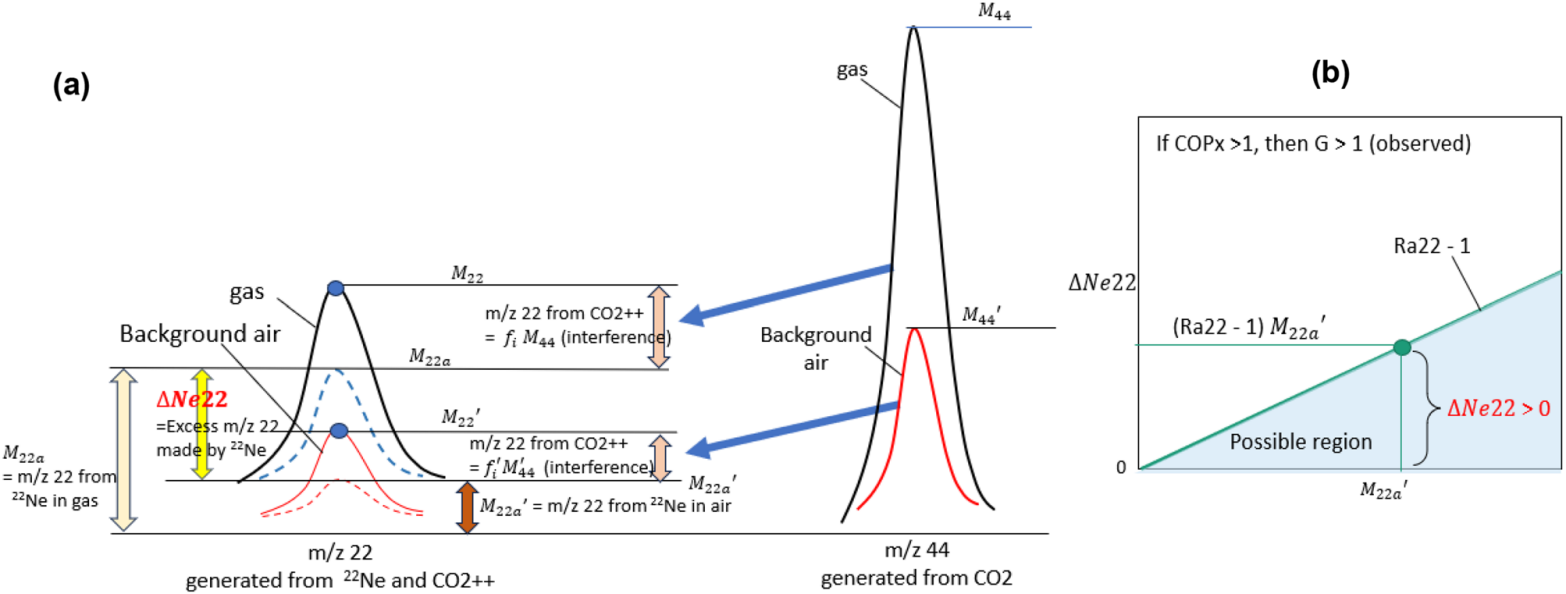
Graphical expression of $\Delta Ne22$. (**a**) Relation between m/z 22 and m/z 44. (**b**) The real $\Delta Ne22$ is between 0 and $\text{(Ra22 - 1) }{{\text{M}}}_{22{\text{a}}}\mathrm{^{\prime}}$ for Ra22 > 1.

Since the m/z 22 signal is generated from ^22^Ne and CO_2_++ (ionization of CO_2_ in MS), the measured m/z 22 signal is the sum of those from ^22^Ne and those from CO_2_++ for gas sample and background air, which can be written as

1 $$\begin{gathered} {\text{Gas}}: M_{22} = f_{i} M_{44} + M_{22a} \hfill \\ {\text{Air}}: M_{22}^{\prime } = f_{i} M_{44}^{\prime } + M_{22a} , \hfill \\ \end{gathered}$$

where *M*_*22 *_: measured m/z 22 signal of gas, *M*_*22a *_: m/z 22 signal contributed from 22Ne, *M*_*44 *_: measured m/z 44 signal of gas, *f*_*i*_: ionization factor of MS, *M*_*22*_*′ *: measured m/z 22 signal of background air,* M*_*22a*_*′ *: m/z 22 signal of background air contributed from ^22^Ne, *M*_*44*_*′ *: measured m/z 44 signal of background air. We can define the excess m/z 22 signal made by ^22^Ne in gas sample as, referring to Fig. 9a,

2 $$\Delta Ne22 = M_{22a} - M_{22a}^{\prime} = {\text{excess m}}/{\text{z 22 signal generated by excess}}^{{{22}}} {\text{Ne}}$$

Combining Eqs. (1) and (2), and the observation of G > 1 in gases from reactors having excess heat, we obtain the following relation:

3 $$G = \frac{{M_{22} - M_{22a} }}{{ M_{22}- M_{22a}^{\prime} {\text{Ra}}22}} > 1$$

where Ra22 = m/z 22 (gas) ÷ m/z 22(air). From Eq. (3), we obtain *M*_*22a*_ < *M*_*22a*_*′* Ra22 and the following relations:

4 $$\Delta Ne22 = M_{22a} - M_{22a}^{\prime} ;\;\frac{\Delta Ne22}{{M_{22a}^{\prime } }} = \frac{{M_{22a} }}{{M_{22a}^{\prime } }} - 1 < {\text{Ra}}22 - 1$$

We finally obtain a relation for excess m/z 22 signal made by excess ^22^Ne in gas sample:

5 $$\Delta Ne22 < {\text{(Ra22 }} - {1) }M_{22a}^{\prime }$$

Since Ra22 is experimentally found to lie between 1.5 and 60.9 for gases from reactors having excess heat as shown in Fig. 8, $(\text{Ra22 - 1})$ > 0 always and $\Delta Ne22$ is shown greater than zero. This proves the presence of ^22^Ne in gases from reactors having excess heat. The real $\Delta Ne22$ lies between 0 and $(\text{Ra22 - 1})\, {M}_{22a}^{\prime}$ as shown in Fig. 9b.

### Proof of ^***22***^Ne presence from isotope-ratio K24a and using CO_***2***_absorber

If CO_2_ in gas sample is absorbed first by Ca(OH)_2_ before entering MS, m/z 22 signal interference from CO_2_++ can be eliminated. ^22^Ne can be identified using the isotope ratio K24a defined as the ratio of R24 with (denoted as “Y”) and without (denoted as “n”) CO_2_ absorption: K24a = R24(Y)/R24(n) where R24(gas) = m/z 22(gas) ÷ m/z 44(gas).

For pure CO_2_ gas, the measured K24a(CO_2_) = 0.96. We can then identify the presence of ^22^Ne simply from K24a > 0.96. It is seen from Table 3 that K24a are greater than 0.96 (maximum 1.49) in all the gases from reactors having excess heat. This confirms the presence of ^22^Ne in gases from reactors having excess heat.

**Table 3:** Using isotope ratios K24a and K22a to identify the presence of ^22^Ne.

									Internal standard method (ISM) based on m/z 40		
Gas sample	Reactor	COPx	CO2 absorption	m/z 22	m/z 40	m/z 44	R24 = m/z 22 ÷ m/z 44	K24a = R24(Y)/R24(n)	I22 = ratio 22/40	I44 = ratio 44/40	K22a = I22(Y)/I22(n)
Pure CO_2_	Reference gas	1.0 (no excess heat)	n	4.44E−06	4.20E−07	4.62E−04	9.61E−03	0.96	10.6	1100.0	0.0060
			Y	8.26E−08	1.31E−06	8.93E−06	9.25E−03		0.0631	6.817	
Tube16	DHX-2B	1.20	n	2.33E−09	8.73E−07	1.99E−07	1.17E−02	**1.07**	0.0027	0.228	**0.55**
			Y	9.33E−10	6.37E−07	7.47E−08	1.25E−02		0.0015	0.117	
Tube39	VCS-NTU	1.54	n	2.94E−08	1.63E−05	4.42E−06	6.65E−03	**1.49**	0.0018	0.271	**1.86**
			Y	4.93E−08	9.35E−06	4.97E−06	9.92E−03		0.0053	0.532	
Tube42	nDHX-2BB	1.22	n	1.55E−07	9.78E−06	1.68E−05	9.21E−03	**1.03**	0.0158	1.717	**0.21**
			Y	3.23E−08	9.92E−06	3.40E−06	9.49E−03		0.0033	0.343	
Tube44	nDHX-2BB	1.16	n	7.75E−08	1.19E−05	9.98E−06	7.77E−03	**1.14**	0.0065	0.839	**0.48**
			Y	4.10E−08	1.30E−05	4.62E−06	8.87E−03		0.0032	0.355	
Tube46	nDHX-2BB	1.15	n	4.87E−08	1.02E−05	5.52E−06	8.82E−03	**1.10**	0.0048	0.541	**0.65**
			Y	3.10E−08	9.93E−06	3.20E−06	9.69E−03		0.0031	0.322	
							K24a > 0.96 reveals ^22^Ne presence		K22a >> 0.006 reveals ^22^Ne presence		

Significant values are in bold.

We can also use another isotope ratio K22a defined as the ratio of I22 with (Y) to without (n) CO_2_ absorption: K22a = I22(Y)/I22(n) where I22 = m/z 22(gas) ÷ m/z 40(gas). The results in Table 3 shows that K22a in all gases is much larger than the pure CO_2_ reference (0.0060). This confirms the presence of ^22^Ne.

## Discussions

In the present study, we have verified using mass spectrometry that ^22^Ne and CO_2_ are produced in water when COP_x_ > 1.05 (having excess heat). The question remaining is how ^22^Ne and CO_2_ is produced. Since this is an unsettled area of science, we can only conjecture some possibilities based on the observed phenomena in various reactors. To find the pathway to ^22^Ne gas production, various possible nuclear reactions could be assumed. In our judgement however, the reaction starts with an interaction between ^1^H and ^16^O, producing ^17^O^14,15^. Two possible reactions for ${}_{8}{}^{17}O$ are proposed as follows:

6 $${}_{1}^{1} H + e^{ - } + \overline{{v_{e} }} + {}_{8}^{16} O \to \blacksquare \to {}_{8}^{17} O$$

7 $${}_{1}^{1} H + e^{ - } + {}_{8}^{16} O \to \blacksquare \to {}_{8}^{17} O + v_{e}$$

where ${}_{1}{}^{1}H$ represents a proton (p), $\overline{{v }_{e}}$ is the ultra-low energy anti-neutrino which can be produced from water cavitation^16^. The ‘black box’ in Eqs. (6) and (7) represents an unknown in detail mechanism^15^. The reaction of ${}_{8}{}^{17}O$ and ${}_{8}{}^{17}O$ then produces ^22^Ne and ^12^C^14,15^:

8 $${}_{8}^{17} O + {}_{8}^{17} O \to \blacksquare \to {}_{6}^{12} C + {}_{10}^{22} Ne$$

This is what we have detected, ^22^Ne in gases from reactors having excess heat.

Since ^12^C cannot exist in monoatomic form, it is converted into CO_2_ through chemical reaction with ^16^O atoms from water, as follows:

9 $${}_{6}^{12} C + 2 {}_{8}^{16} O \to CO_{2}$$

This is what we have detected, CO_2_ in gases from reactors having excess heat.

The difference between Eqs. (6) and (7) is the involvement of neutrino ${v}_{e}$ and anti-neutrino $\overline{{v }_{e}}$. In Eq. (6), $\overline{{v }_{e}}$ acts over a wide area to allow the 4-particle reaction with ${}_{1}{}^{1}H, {e}^{-}, {}_{8}{}^{16}O$. That is, the low-energy anti-neutrino is the cause of increased probability of the 4-particle reaction. In Eq. (7), ${v}_{e}$ is the product of the 3-particle reaction which may have high energy. Since Eq. (7) involves only three-particles merging, it might be more likely to take place from the point of view of the particle collision probability if there is some way to force these particles into a confined zone.

The reaction Eq. (6) is exothermic and emits no radiation. A Geiger–Müller counter (JD-3001) monitoring radiation near the reactors during test, never showed a significant emission level above background, measured at 0.4 μSv/h maximum which is about two times of background or 0.4% occurrence. This seems favor the reaction Eq. (6).

In Ref.^15^, it is proposed from nuclear physics that neutrinos and anti-neutrino pair at low energies can be formed during inelastic collisions of particles (electrons, ions, neutral atoms) during their thermal motion. Temperatures known to be produced in cavitation processes may exceed the calculated threshold in Ref.^15^ for low energy neutrino and anti-neutrino pair production. The question then arises as to how the 4 particles (${}_{1}{}^{1}H+{e}^{-}+\overline{{v }_{e}}+{}_{8}{}^{16}O)$ in Eq. (6) can merge simultaneously. However due to the large de Broglie wavelength of low energy neutrinos, they could probably interact over an area big enough to include all the particles necessary for reaction Eq. (6) to take place.

Since the mechanism of nuclear reactions is not the focus of our research, we can only put forward intuitive and vague speculations with respect to our experimental observations. The present conjecture, reaction (6) or (7), is just two possibilities.

Another question raised is that “are there extra ^17^O and ^12^C to produce other compounds ?” Both ^17^O and ^12^C does not exist in monoatomic form. They will form compounds with other elements. This means that ^12^C and isotope ^17^O may be the intermediate.

Nuclear transmutation in ruptured copper pipe with large increases of C and O contents is one of the possible outcomes and has been observed in SEM/EDS^11^. Besides this, we found that three isotopes may be the outcomes: H_2_O-17(heavy-oxygen water), isotope O_2_ (^16^O–^17^O), and isotope CO_2_ (^12^C–^16^O–^17^O). This can be identified from isotope ratio analyses in m/z 19, 33 and 45 signals.

### Finding of H_***2***_O-17 (heavy-oxygen water)

The isotope ratio R198 using internal standard m/z 40 is defined as R198 = L198(gas)/L198(air) where L198(gas) = I19(gas)/I18(gas), L198(air) = I19(air)/I18(air), and I18(gas) = m/z 18(gas) ÷ m/z 40(gas), I18(air) = m/z 18(air) ÷ m/z 40(air); I19(gas) = m/z 19(gas) ÷ m/z 40(gas), I19(air) = m/z 19(air) ÷ m/z 40(air).

The m/z 19 signal may be generated from HDO (deuterium water) and isotope H_2_O-17. Since steam (Tube9) has highest content of HDO (deuterium water), the measured R198(steam) = 1.10 is the maximum contribution of HDO to m/z 19. This is used to distinguish H_2_O-17 from HDO.

**Figure 10:**
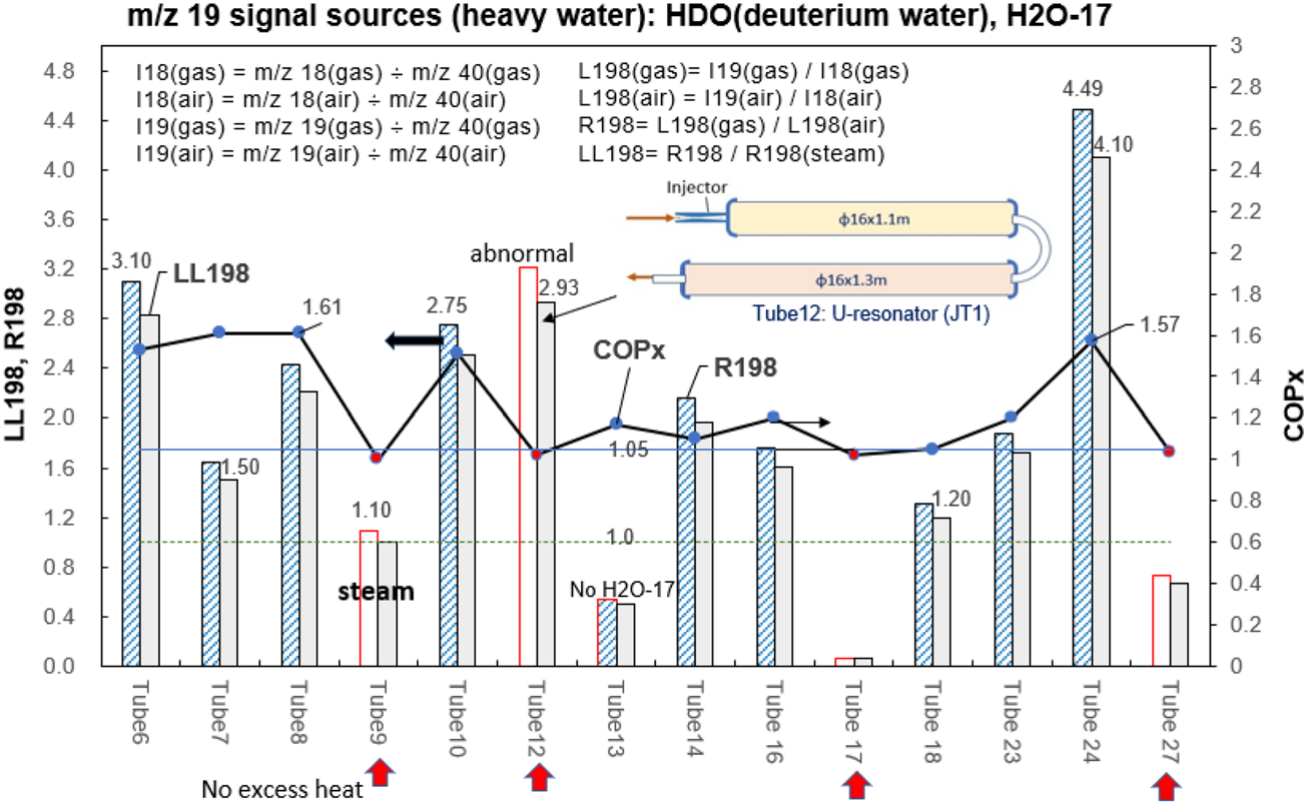
Variation of R198 and LL198 for gas from different reactors. Using isotope ratios R198 and LL198 to distinguish H_2_O-17 from HDO.

LL198 is further defined as LL198 = R198/R198(steam) to provide a criterion to identify the presence of H_2_O-17 when LL198 > 1.0. It is seen from Fig. 10 that LL198 >> 1 appearing in 9 out of 10 gases from reactors having excess heat. This strongly suggests significant contribution to m/z 19 from H_2_O-17, other than HDO. The presence of heavy-oxygen water H_2_O-17 is thus confirmed.

Tube12 gas contains no ^22^Ne and CO_2_ (implying no excess heat), but produces isotope H_2_O-17 as seen from Fig. 10. Tube12 gas was collected from U-resonator (JT1) without excess heat (COP_x_ < 1.05). However, we found that the reactor showed abnormal temperature variations. It involves some other peculiar phenomena and needs further studies.

### Finding of isotope O_2_*** (***^***16***^***O–***^***17***^***O)***

**Figure 11:**
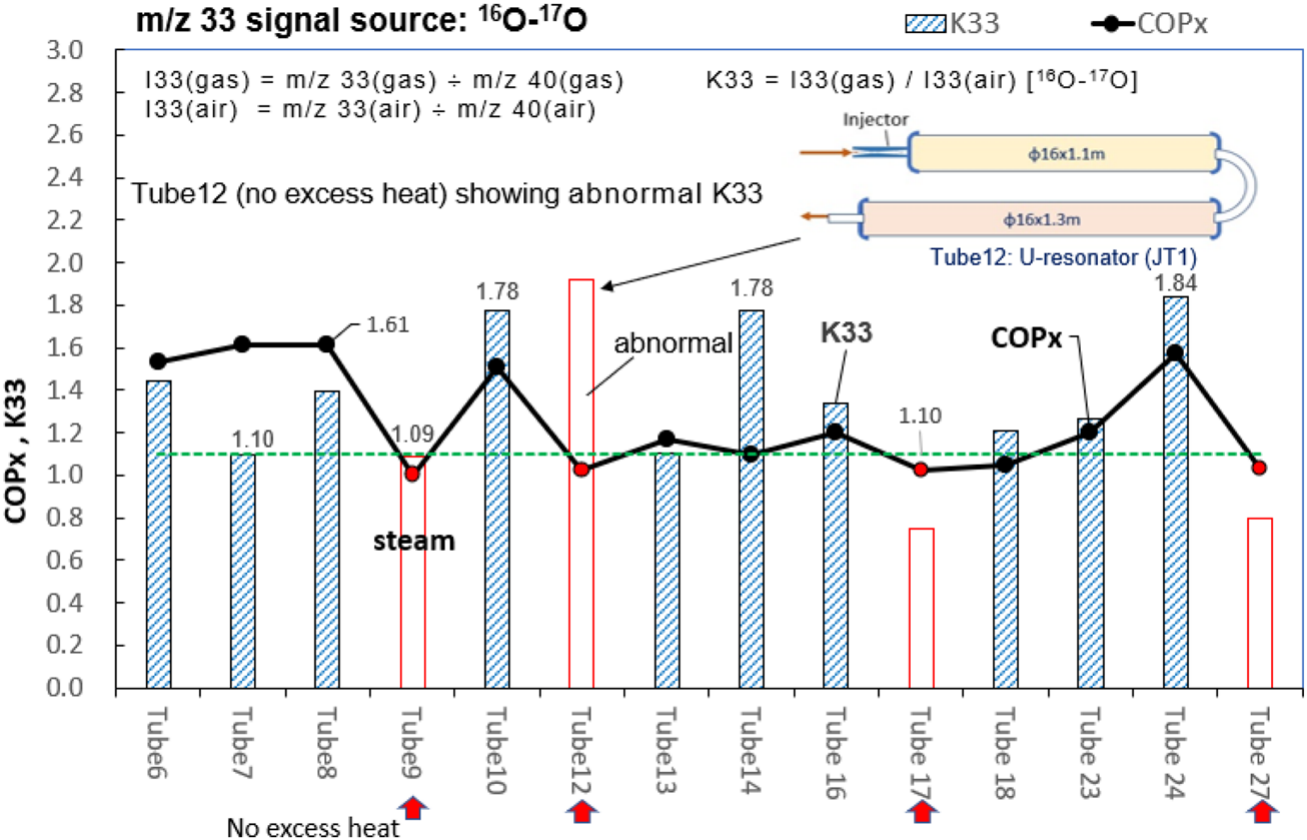
Variation of K33 for gas from different reactors. Using isotope ratio K33 to identify isotope O_2_ (^16^O–^17^O).

To trace isotope O_2_ (^16^O-^17^O), we define the isotope ratio K33 using internal standard m/z 40 as: K33 = I33(gas) / I33(air) where I33(gas) = m/z 33(gas) ÷ m/z 40(gas); I33(air) = m/z 33(air) ÷ m/z 40(air). It is seen from Fig. 11, K33 > 1.1 in all gases from reactors having excess heat. This strongly suggests the presence of isotope O_2_ (^16^O-^17^O). Again, Tube12 contains no ^22^Ne and CO_2_ gas but isotope O_2_ (^16^O-^17^O) is present as seen from Fig. 11. This implies that some other peculiar phenomena may take place in U-resonator (JT1) and is worth for further investigations.

### Finding of isotope O_2_ (^16^O–^17^O)

**Figure 12:**
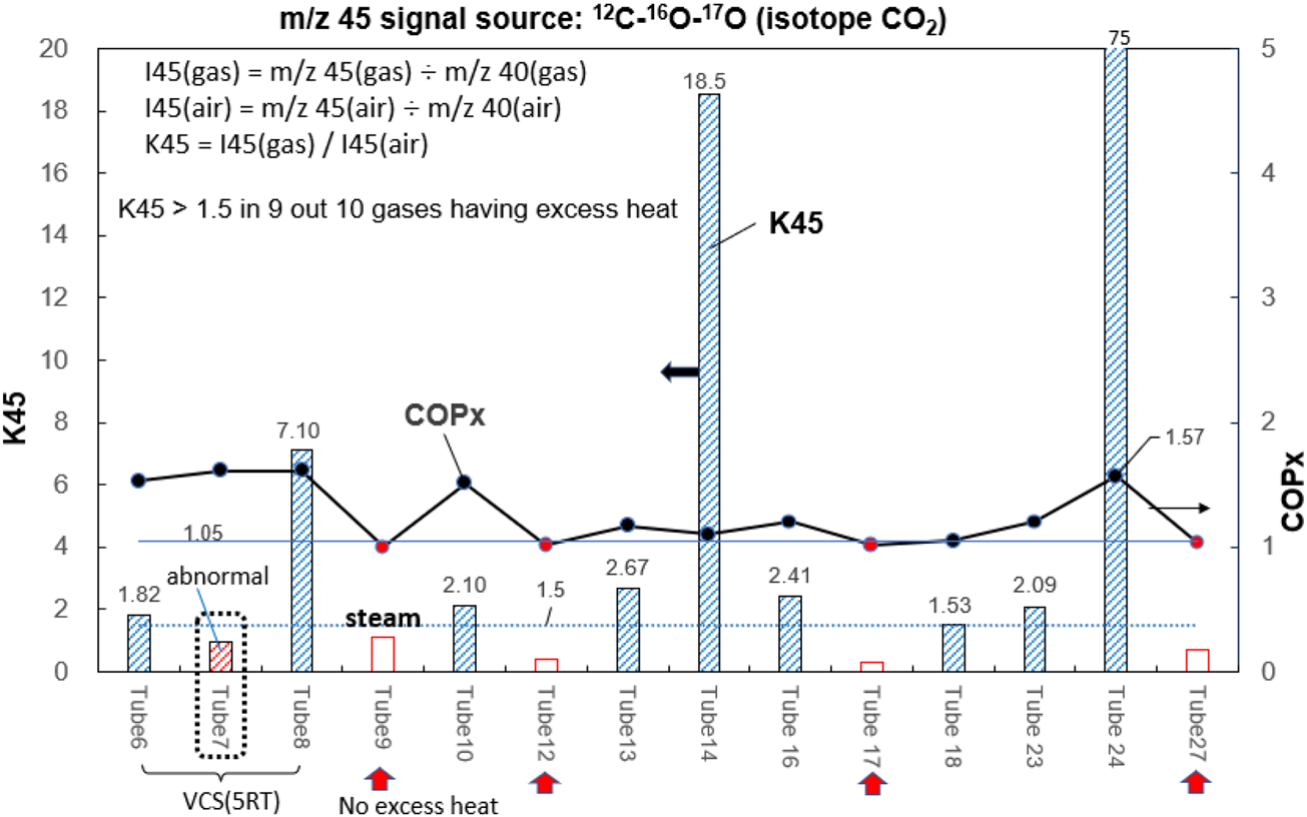
Variation of K45 for gas from different reactors. Using isotope ratio K45 to identify isotope CO_2_ (^12^C–^16^O–^17^O).

To trace isotope CO_2_ (^12^C-^16^O-^17^O), we define the isotope ratio K45 using internal standard m/z 40: K45 = I45(gas)/I45(air) where I45(gas) = m/z 45(gas) ÷ m/z 40(gas); I45(air) = m/z 45(air) ÷ m/z 40(air). It is seen from Fig. 12, K45 > 1.5 takes place in gases from reactors having excess heat. High value of K45 (2.1–75) strongly suggests the presence of isotope CO_2_ (^12^C–^16^O–^17^O).

### Conditions for presence of isotopes H_2_O-17 (heavy-oxygen water), CO_***2***_(^12^C–^16^ O–^17^O), O_2_^16^O–^17^O)

The presence of isotopes O_2_ and CO_2_ relies on the extra quantities of ^12^C and ^17^O resulting from the main nuclear reactions. Sometimes they may not appear due to no extra ^12^C or ^17^O. Tube7 in Fig. 6 may be the case. Nevertheless, the above results cannot deny the possible presence of isotopes O_2_, CO_2_ and H_2_O-17 (heavy-oxygen water) in gases from reactors having excess heat.

Finally, it should be noted that the reactors tested in the present study were under early developing stage. Although their performance has not been optimized yet, the anomalous gas produced from reactors having excess heat is always present. The reactor is still being optimized to improve the performance. A higher concentration of non-condensable gases may be expected. This will make the MS analysis easier.

## Conclusion

Cavitation may induce implosion of water vapor bubbles using various techniques^1–10^. In the previous study, we found that the heat exchange process in multiple-pipe heat exchanger produces anomalous excess heat and nuclear transmutation^11^. Recently, we have tested another 8 reactors and found that they also produce non-condensable gas. We suspected that ^22^Ne and CO_2_ may exist and is from nuclear reactions of water.

Fourteen gas samples were collected from eight reactors to perform mass spectrometry carefully using various methods. Two different methods for the identification of CO_2_ were employed, while three different methods are employed for ^22^Ne. All the results confirm that ^22^Ne and CO_2_ do exist in gas samples from reactors having excess heat.

In answering the question “how ^22^Ne and CO_2_ is produced in water”, we conjecture a possible mechanism and find out that ^12^C and isotope ^17^O may be the intermediate. They possibly produce some other isotope compounds in gas from reactors having excess heat. Using isotope ratio analysis, we find out that they are H_2_O-17 (heavy-oxygen water), isotope O_2_ (^16^O–^17^O), and isotope CO_2_ (^12^C–^16^O–^17^O).

We also find that the reactions Eqs. (6)–(8) are the most-probable reactions whose output gas contents coincide with our observations—detected CO_2_ and isotopes ^22^Ne, H_2_O-17, CO_2_ (^12^C–^16^O–^17^O) and O_2_ (^16^O–^17^O), although the detailed mechanism is not known. This needs further basic research. Since the chances of getting all the present conclusion is so remote, particularly the presence of ^22^Ne without ^20^Ne or ^21^Ne, this work may lead to a new research topic in nuclear science and energy technology if true.

## Data Availability

All data generated or analyzed during this study are included in this published article and are available from the corresponding author on reasonable request.
